# Targeted Drug Discovery for Pediatric Leukemia

**DOI:** 10.3389/fonc.2013.00170

**Published:** 2013-07-08

**Authors:** Andrew D. Napper, Venita G. Watson

**Affiliations:** ^1^High-Throughput Screening and Drug Discovery Laboratory, Nemours Center for Childhood Cancer Research, A.I. duPont Hospital for Children, Wilmington, DE, USA

**Keywords:** drug discovery, targeted therapy, leukemia, molecular target, medicinal chemistry, lead optimization

## Abstract

Despite dramatic advances in the treatment of pediatric leukemia over the past 50 years, there remain subsets of patients who respond poorly to treatment. Many of the high-risk cases of childhood leukemia with the poorest prognosis have been found to harbor specific genetic signatures, often resulting from chromosomal rearrangements. With increased understanding of the genetic and epigenetic makeup of high-risk pediatric leukemia has come the opportunity to develop targeted therapies that promise to be both more effective and less toxic than current chemotherapy. Of particular importance is an understanding of the interconnections between different targets within the same cancer, and observations of synergy between two different targeted therapies or between a targeted drug and conventional chemotherapy. It has become clear that many cancers are able to circumvent a single specific blockade, and pediatric leukemias are no exception in this regard. This review highlights the most promising approaches to new drugs and drug combinations for high-risk pediatric leukemia. Key biological evidence supporting selection of molecular targets is presented, together with a critical survey of recent progress toward the discovery, pre-clinical development, and clinical study of novel molecular therapeutics.

## Pediatric Leukemia: The Need for Targeted Therapies

**Figure 1:**
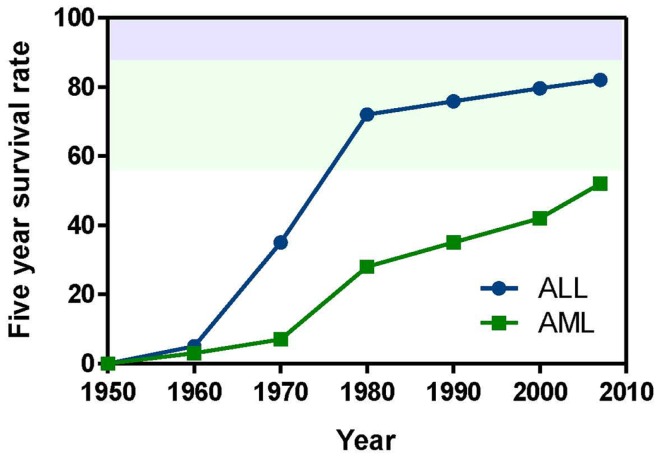
**Pediatric leukemia survival trends from 1950 to the present**. Data obtained from Kersey (1997) and Pui et al. (2011b).

This review focuses on pediatric leukemia, both acute lymphoblastic leukemia (ALL) and acute myeloid leukemia (AML), which together account for approximately one third of childhood cancers. ALL is primarily a childhood disease, whereas AML is much more prevalent in adults (National Cancer Institute, 2010). However, due to its poorer prognosis, AML accounts for as many childhood deaths per year as ALL. Aggressive treatment of children with combinations of cytotoxic drugs has raised the 5-year survival rate for pediatric ALL above 80%. Given that less than one child in 10 survived ALL in the early 1960s, this is a remarkable achievement. However, this progress has come at the cost of severe toxicity, and nearly 20% of ALL cases either do not respond or relapse following treatment. The 5-year survival rate for pediatric AML is under 60%. AML that arises following treatment for another malignancy (therapy-related AML) is particularly difficult to cure (Kurmasheva and Houghton, 2007; Pui et al., 2011b). Figure 1 highlights how progress has slowed following a dramatic improvement in survival from the 1950s–1980s.

In the past 30 years, few new anti-cancer agents have been approved for use in pediatric patients. Survival in very high-risk patient groups remains poor despite the most intensive cytotoxic therapies. Rationally designed drugs have benefited adults with cancer, but except in the case of BCR-ABL positive leukemias, targeted therapies have so far done little to improve pediatric outcomes. However, there are encouraging signs that this situation is about to change. Study of the mechanistic basis for pediatric cancer has begun to uncover defined molecular targets, raising the possibility of treatment by specifically designed drugs (Mackall, 2011). In most cases of pediatric leukemia, the genetic profile is well-defined, and patients at high risk of poor response to treatment may be readily identified. However, this characterization of leukemia by subtype generally has not led to much improvement in outcome, due to an inability to develop effective new drugs because of a lack of understanding of the biological mechanisms connecting specific genetic abnormalities and leukemia progression (Pui et al., 2011a). Readers are referred to recent reviews (Brandwein, 2011; Pui et al., 2011b) for current treatment regimens and clinical outcomes. A notable exception to the dismal lack of success in treating high-risk leukemia in children has been the remarkable efficacy of the BCR-ABL kinase inhibitor imatinib (Gleevec) in Philadelphia-positive (Ph^+^) ALL (Barr, 2010). In recent years other points of intervention have been identified that may respond to targeted drugs. This review highlights the biological evidence pointing to novel specific molecular targets and the progress of drug discovery efforts based on this evidence, especially for some of the most intractable high-risk subtypes of ALL and AML.

## Molecularly Targeted Drug Discovery and Development

**Figure 2:**
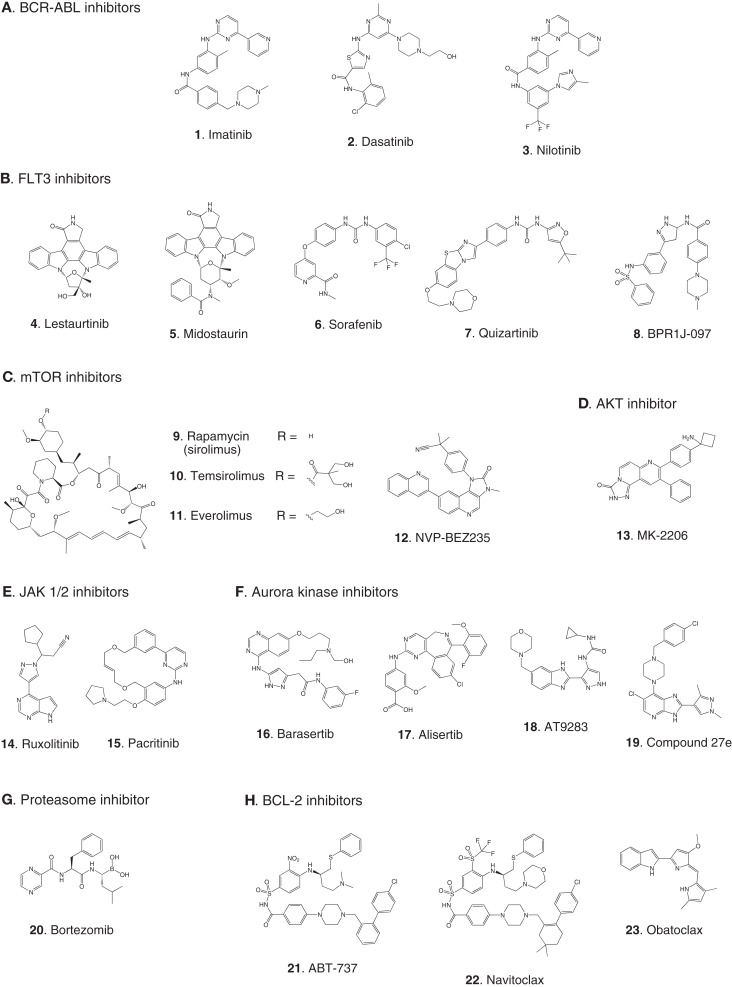
**Compounds targeting inhibition of cell proliferation or induction of apoptosis**. Numbers in bold type are used to refer to compounds in this figure from the main text, Figure 4, and Table 1. **(A)** BCR-ABL inhibitors, **(B)** FLT3 inhibitors, **(C)** mTOR inhibitors, **(D)** AKT inhibitor, **(E)** JAK1/2 inhibitors, **(F)** aurora kinase inhibitors, **(G)** proteasome inhibitor, **(H)** BCL-2 antagonists.

**Figure 3:**
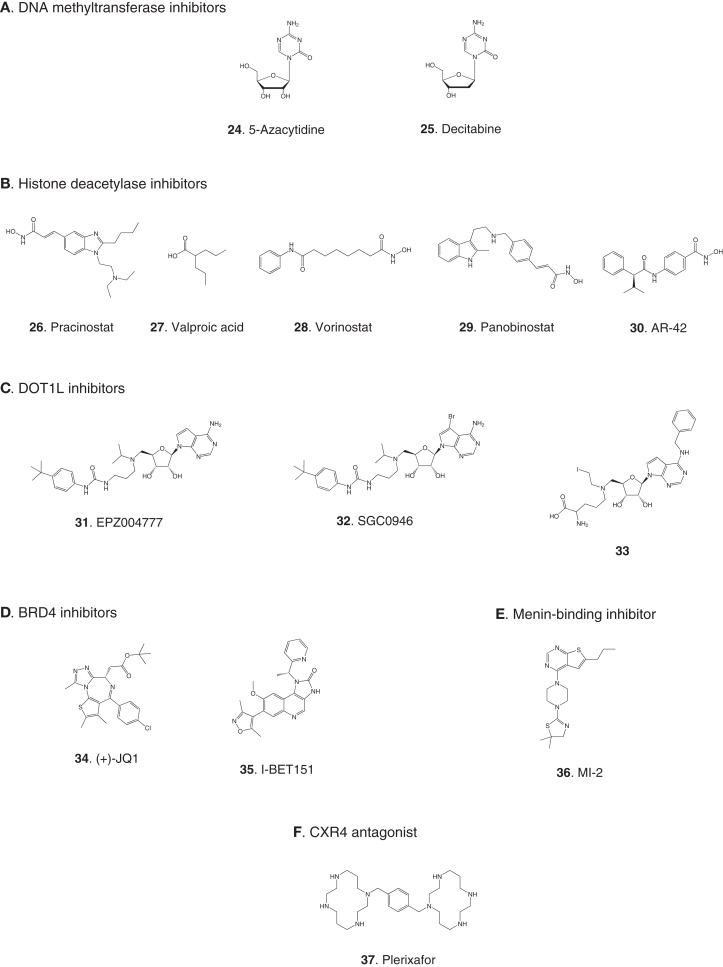
**Compounds targeting transcription or stem cell homing**. Numbers in bold type are used to refer to compounds in this figure from the main text, Figure 5, and Table 1. **(A)** DNA methyltransferase inhibitors, **(B)** histone deacetylase inhibitors, **(C)** DOT1L inhibitors, **(D)** BRD4 inhibitors, **(E)** Menin-binding inhibitor, **(F)** CXCR4 antagonist.

The approval of imatinib in 2001 to treat chronic myeloid leukemia (CML) and its remarkable success rate – 90% 5-year survival – spurred efforts to develop additional molecularly targeted therapies for cancer and other diseases. The majority of this effort has been directed toward adult disease, but an increased understanding of the biology of pediatric cancer has fostered progress toward targeted treatment of some of the most intractable cases (Brown et al., 2009). The NCI lists 207 treatment clinical trials ongoing for pediatric ALL and 188 for AML. (Note that the total number is substantially less than 395 because trials for both ALL and AML are counted twice.) Of these, 26 (13 both ALL and AML, 10 ALL and 3 AML) are testing molecularly targeted agents (National Cancer Institute, 2013). Numerous compound series are in earlier stages of lead optimization and pre-clinical development. This review is organized by broad disease targeting mechanisms, and further subdivided by protein target classes. Numbers in bold type in parentheses after compound names may be used to locate compounds in Table 1, which summarizes the current roster of clinical candidates and references the clinical protocol IDs (National Cancer Institute, 2013). The bold-type compound numbers also will help the reader locate molecular structures depicted in Figures 2 and 3. Sections “Cell Proliferation and Apoptosis, Transcription, and Chemokine Receptors and Stem Cell Homing” cover candidates in clinical trials and highlight the most recent and innovative examples of compounds in early discovery and pre-clinical development. Several recent reviews cover specific targets and pathways, and the reader is referred to these for more comprehensive coverage. Although a majority of the agents under development are aimed at arrest of cell proliferation and induction of apoptosis, substantial work is also focused on modulation of transcription, and homing of stem cells to the bone marrow.

**Table 1:** **Compounds currently in clinical trials as targeted drugs in pediatric ALL or AML**.

Target class	Target	Section	Compound number*	Compound name	Disease	Trial phase	Clinical protocol ID
Protein tyrosine kinases	BCL-ABL	BCR-ABL	2	Dasatinib	ALL	II	CA180-226
							2008-002260-33
							NCT00777036
					ALL	II	CA180-372
							2011-001123-20
							AALL1122
	BCL-ABL	BCR-ABL	3	Nilotinib	ALL	I	CAMN107A2120
							2010-018419-14
							NCT01077544
	FLT3	FLT3 and Other RTKs	4	Lestaurtinib	ALL	III	CDR0000573996
							COG-AALL0631
							NCT00557193
	FLT3	FLT3 and Other RTKs	5	Midostaurin	ALL/AML	I/II	CPKC412A2114
							2008-006931-11
							NCT00866281
	FLT3	FLT3 and Other RTKs	6	Sorafenib	AML	III	NCI-2011-02670
							COG-AAML1031
							NCT01371981
					ALL/AML	I	RELHEM
							NCT00908167
	FLT3	FLT3 and Other RTKs	7	Quizartinib	ALL/AML	I	T2009-004
							NCT01411267
Protein serine/threonine kinases	mTOR	mTOR	9	Rapamycin	ALL	II	10-007444
							6137-09
							NCT01162551
					ALL/AML	I	CHP-755
							CHP-IRB-2002-12-3086
							NCT00068302
	mTOR	mTOR	10	Temsirolimus	ALL	I	T2008-004
							NCT01614197
					ALL	I	NCI-2011-02679
							COG-ADVL1114
							NCT01403415
	mTOR	mTOR	11	Everolimus	ALL	I	11-237
							NCT01523977
	AKT	AKT	13	MK-2206	ALL/AML	I	COG-ADVL1013
							NCT01231919
	JAK1/2	JAK1/2	14	Ruxolitinib	ALL/AML	I	CDR0000680970
							COG-ADVL1011
							NCT01164163
	Aurora A	Aurora Kinases	17	Alisertib	ALL/AML	II	CDR0000680512
							COG-ADVL0921
							NCT01154816
	Aurora A/B	Aurora Kinases	18	AT9283	ALL/AML	I	CRUK-CR0708-12
							EUDRACT-2009-016952-36
							NCT01431664
Proteases	Proteasome	Proteasome	20	Bortezomib	AML	III	NCI-2011-02670
							COG-AAML1031
							NCT01371981
					ALL	II	2008LS113
							MT2008-33R
							NCT01312818
					ALL	II	CDR0000638413
							COG-AALL07P1
							NCT00873093
Anti-apoptotic proteins	Bcl-2	Anti-Apoptotic Protein BCL-2	23	Obatoclax	ALL/AML	I	COG-ADVL0816
							NCT00933985
DNA methyltransferases	Various	DNA Methyltransferases	24	5-Azacytidine	AML	II	J1240
							P01CA015396
							NCT01700673
Histone deacetylases	Various	Histone Deacetylases	28	Vorinostat	ALL/AML	II	NCT-2007-11-02-1004
							NCT01422499
	Various	Histone Deacetylases	29	Panobinostat	ALL/AML	I	T2009-012
							NCT01321346
	Various	Histone Deacetylases	30	AR-42	AML	I	OSU-11130
							NCI-2013-00122
							NCT01798901
Chemokine receptors	CXCR4	Chemokine Receptors and Stem Cell Homing	37	Plerixafor	ALL/AML	I	POETIC Plerixafor NCT01319864

*Refers to compound number in text and in Figures 2 and 3.

### Cell proliferation and apoptosis

**Figure 4:**
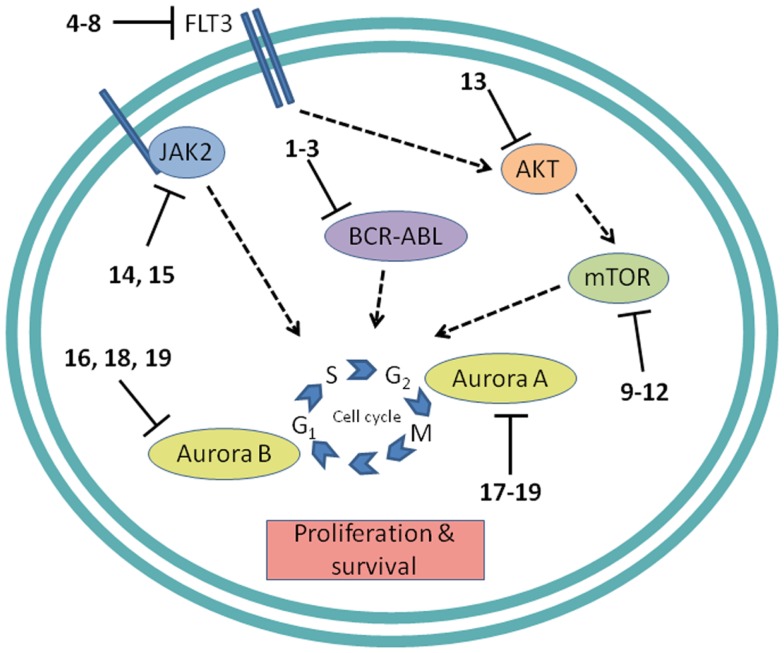
**Cell proliferation targets**. Numerous interconnections between targets and other cellular proteins are omitted for clarity. Numbers in bold type refer to inhibitors shown inFigure 2.

Development of protein kinase inhibitors as therapeutic agents for adult cancer has been an area of intense effort for many years. Likewise, certain protein kinases in pediatric acute leukemia have been targeted because they mediate cell signaling that promotes growth and resists apoptosis. The most notable success has been the inhibition of BCR-ABL, while much attention continues to be focused on the receptor tyrosine kinase (RTK) FMS-like tyrosine kinase-3 (FLT3), the mTOR/AKT pathway, JAK/STAT signaling, and control of mitosis by aurora kinases (Figure 4). In addition to protein kinases, the proteasome, and the anti-apoptotic protein BCL-2 are being targeted for inhibition to induce apoptosis in pediatric leukemia.

#### Protein tyrosine kinases

##### BCR-ABL

The first example of a molecularly targeted therapy for cancer was imatinib (**1**) (Gleevec), an inhibitor of the ABL tyrosine kinase activity of the fusion protein BCR-ABL. This was initially developed to treat CML in adults, but has since been shown to be highly effective against Ph^+^ ALL in children, which also harbors the BCL-ABL fusion (Schultz et al., 2009; Barr, 2010), and it was recently approved by the FDA for this indication (Food and Drug Administration, 2013). Since use of imatinib in children began, the 5-year survival rate for pediatric Ph^+^ ALL has more than doubled to 80%. However, the appearance of mutant forms of the kinase domain of BCR-ABL has led to resistance to imatinib (Hunger, 2011). Therefore, new series of inhibitors have been developed to target mutant forms of the enzyme as well as the wild type. Dasatinib (**2**) is currently in two Phase II trials for pediatric Ph^+^ ALL. Nilotinib (**3**) binds 30-fold more tightly than imatinib to BCL-ABL in resistant leukemia cells and is currently in Phase I pediatric trials. However, neither dasatinib nor nilotinib are effective against so-called gatekeeper mutations, notably T315I. This shortcoming has been overcome by structure-based design of ponatinib, which gave encouraging Phase I results in adult CML and Ph^+^ ALL (Cortes et al., 2012). A Phase II trial of ponatinib in Ph^+^ ALL is due to start soon, but enrollment is limited to adult patients. (See Figure 2A for structures of the compounds discussed in this section.)

##### FLT3 and other RTKs

Receptor tyrosine kinases play an important signal transduction role in acute leukemia. The *FLT3* gene is mutated in a significant proportion of high-risk pediatric ALL and AML; activating FLT3 mutations occur in 22% of AML and 18% of MLL-rearranged ALL. AML harboring *FLT3* internal tandem duplications (*FLT3*-ITD) is associated with an especially poor prognosis (Brown et al., 2006; Stubbs and Armstrong, 2007; Swords et al., 2012). FLT3 mutations promote leukemia survival and proliferation and block cell differentiation (Sexauer et al., 2012). So far, FLT3 inhibitors have shown limited effectiveness as single agent therapies, due to several confounding factors: low plasma levels of compounds due to poor pharmacokinetics, limited efficacy due to high levels of competing FLT3 ligand (FL), and point mutations arising in the inhibitor binding pocket (Grunwald and Levis, 2013). Intense effort is focused on optimizing clinical response through design of improved compounds, adjustment of dosage of conventional chemotherapy to attenuate FL levels, and development of combination therapies. Currently, compounds are in clinical trials as therapies to treat MLL-rearranged leukemia (ALL and AML) and AML harboring *FLT3*-ITD (Daver and Cortes, 2012). The staurosporine analog Lestaurtinib (**4**) is being tested in MLL-rearranged infant ALL in a multicenter Phase III trial in combination with conventional chemotherapy. A Phase I/II dose-ranging trial of Midostaurin (**5**), another analog of the natural product staurosporine, is ongoing in pediatric ALL and AML. Other structural classes are also being tested. Sorafenib (**6**), originally developed as a B-RAF inhibitor, also inhibits FLT3 and is selectively cytotoxic to leukemia cells harboring FLT3 mutations. It is currently in a Phase III trial in AML, as well as a Phase I trial in combination with conventional chemotherapy. The most potent FLT3 inhibitor in cells harboring *FLT3*-ITD is quizartinib (**7**), with an IC_50_ of 1 nM (Kampa-Schittenhelm et al., 2013). It is currently in a Phase I trial in relapsed or refractory ALL and AML. A recent report describes BPR1J-097 (**8**), a FLT3 inhibitor in pre-clinical development with selectivity and *in vivo* pharmacokinetics that are apparently superior to those of earlier compound classes (Lin et al., 2012).

In two very recent reports evidence of the involvement of other RTKs in AML offers the possibility of additional molecular targets: AXL was shown to activate FLT3 in AML (Park et al., 2013), and siRNA knockdown of *Mer* reduced colony formation and increased survival in mice (Lee-Sherick et al., 2013). (See Figure 2B for structures of the compounds discussed in this section.)

#### Protein serine/threonine kinases

##### mTOR

The PI3 kinase/AKT/mTOR pathway is activated in many acute leukemias, and promotes leukemia cell survival and proliferation (Barrett et al., 2012). Accordingly, mTOR inhibitors are being tested as single agents and in combination with existing chemotherapeutics. mTOR is a serine/threonine kinase, but the approach to inhibition of this target has generally differed from that of small-molecule active site-directed inhibitors employed against other protein kinases. Current clinical studies are focused on the macrolide natural product rapamycin and analogs, which inhibit mTOR once complexed with FK-binding protein 12 (FKBP12). Rapamycin (**9**) (sirolimus) is in a Phase II ALL trial in combination with methotrexate, and in Phase I studies in ALL and AML as a single agent. Temsirolimus (**10**), an analog of sirolimus, is being tested in combination with etoposide and cyclophosphamide in a Phase I ALL trial, and an ALL trial in combination with several other chemotherapeutic agents is due to start soon. Everolimus (**11**), another analog of sirolimus, is marketed for several adult cancer indications. It is currently in a Phase I ALL trial in combination with induction chemotherapy. A promising alternative to macrolide inhibition of the mTOR-FKBP12 complex is the development of dual PI3 kinase/mTOR inhibitors (Martelli et al., 2012). The ATP-binding pockets in the catalytic sites of PI3 kinase and mTOR are structurally similar, allowing the development of compounds that inhibit both enzymes. The dual PI3 kinase/mTOR inhibitor NVP-BEZ235 (**12**) was originally developed for the treatment of solid tumors (Maira et al., 2008), but it has recently shown potent activity in ALL, and synergy with chemotherapeutic agents, even in glucocorticoid-resistant cells (Schult et al., 2012). (See Figure 2C for structures of the compounds discussed in this section.)

##### AKT

AKT is a serine/threonine kinase functionally connected with mTOR complexes that is a central component of signaling through RTKs such as FLT3 (Park et al., 2010). Therefore, AKT inhibition offers an approach to proliferative pathway suppression that is complementary to inhibition of FLT3 or mTOR described above. AKT inhibitors are being evaluated as single agents; MK-2206 (**13**) is in Phase I trials in recurrent or refractory ALL and AML. However, their most promising use could be as potentiators of the efficacy of FLT3 inhibitors. Despite indisputable evidence that FLT3 is a key driver in AML, and the discovery of potent, selective inhibitors, the clinical effectiveness of FLT3 inhibitors has been modest. A significant reason for this appears to be that leukemia stem cells (LSCs) reside in the bone marrow, where they are protected from drug treatment. A high-throughput screen to test investigational drug combinations in a co-culture of FLT-positive AML and stromal cells found that MK-2206 (**13**) synergistically potentiated the cytotoxic effect of the FLT3 inhibitor midostaurin (**5**) (Weisberg et al., 2013).

An unexpected finding in the AKT pathway is that members of the FOXO family of transcription factors enforce differentiation blockade in AML (Sykes et al., 2011). The FOXOs are usually considered to be tumor suppressors that are inhibited by AKT through serine/threonine phosphorylation. However, in approximately 40% of AML, AKT itself is repressed, and the FOXOs are elevated and found to be essential to maintenance of an undifferentiated state. Activation of AKT or deletion of the FOXOs caused myeloid maturation and apoptosis in AML cells, but this effect was countered by a resistance mechanism in which c-JUN was activated, so a combination of FOXO and JNK (c-JUN N-terminal kinase) inhibition may be an effective therapeutic strategy (Sykes et al., 2011). (See Figures 2B,D for structures of the compounds discussed in this section.)

##### JAK1/2

The JAK/STAT pathway is activated in certain high-risk leukemias, especially Ph^+^-like ALL, leading to cell proliferation and resistance to apoptosis (Maude et al., 2012). Activating mutations of JAK kinases are also present in AML (Daver and Cortes, 2012). A Phase I clinical trial is underway to test a selective JAK1/2 inhibitor, ruxolitinib (**14**). The aim is to select patients with refractory or relapsed ALL or AML harboring JAK mutations, although leukemia patient numbers may be limited because the trial also includes patients with solid tumors. JAK inhibitors may also be useful in combination with other targeted therapies. The JAK2/FLT3 inhibitor pacritinib (**15**) demonstrated synergy with the histone deacetylase (HDAC) inhibitor pracinostat (**26**) in mouse AML models (Novotny-Diermayr et al., 2012). This JAK2/HDAC inhibitor synergy may arise due to JAK2 possessing an epigenetic function; in addition to its role in activation of JAK-STAT signaling, JAK2 phosphorylates tyrosine 41 in histone 3 (H3Y41), leading to displacement of heterochromatin protein 1α. JAK inhibitors were also found to potentiate the cytotoxicity of FLT3 inhibitors in a high-throughput screen of investigational drug combinations in a co-culture of AML and stromal cells, suggesting that JAK inhibitors may be effective in overcoming the drug resistance of AML residing in the bone marrow (Weisberg et al., 2012). In a related study, discussed in Section “AKT” above, the same research group observed synergy between AKT and FLT3 inhibition. (See Figures 2E and 3B for structures of the compounds discussed in this section.)

##### Aurora kinases

Aurora kinases A and B are serine/threonine kinases that play a key role in the control of mitosis. Overexpression of these enzymes has been observed in pediatric leukemia and is associated with a poor prognosis (Farag, 2011). Although both enzymes have generated interest as targets, short hairpin RNA knockdown, and corroboration using the selective aurora kinase B inhibitor barasertib (**16**) suggested that antiproliferative and pro-apoptotic effects are mediated predominantly through inhibition of aurora kinase B (Hartsink-Segers et al., 2013). These findings appear to be at odds with clinical progress to date with the selective aurora A inhibitor alisertib (**17**). Alisertib is currently in a Phase II trial that includes refractory and relapsed ALL and AML as well as pediatric solid tumors. Fragment-based drug discovery was used to develop AT9283 (**18**), an inhibitor of aurora kinases A and B and also JAK2. This compound was found to inhibit growth and survival of patient-derived leukemia cells, associated with a reduction in levels of phosphorylated FLT3 and inhibition of downstream effectors such as ERK and MEK (Jayanthan et al., 2013). On the basis of the encouraging pre-clinical results, AT9283 is being tested in relapsed and refractory ALL and AML in a Phase I trial in the UK. A potentially important development is the discovery of compounds that inhibit both FLT3 and aurora kinases; a dual FLT3/aurora kinase inhibitor known simply as compound 27e (**19**) may show enhanced clinical efficacy due to synergy between FLT3 and aurora kinase inhibition (Bavetsias et al., 2012). (See Figure 2F for structures of the compounds discussed in this section.)

#### Proteasome

Bortezomib (**20**) is a highly potent proteasome inhibitor that protects tumor suppressor proteins from degradation. It is approved and marketed for the treatment of multiple myeloma, but it also promises to be effective in combination with other targeted therapies and conventional chemotherapy in leukemia (Niewerth et al., 2013). Combination of bortezomib with the HDAC inhibitor valproic acid (**27**) was effective against B-cell precursor ALL in cell culture and mouse xenograft with or without simultaneous treatment with chemotherapeutic agents. Evidence suggests that the activity of the drug combination is mediated through modulation of NF-κB activity, thereby promoting apoptosis (Bastian et al., 2013). A Phase I trial in pediatric ALL and AML demonstrated the effectiveness of bortezomib as a chemosensitizer in conjunction with chemotherapy, although not as a single agent (Horton et al., 2007). Bortezomib is currently being tested in a Phase III trial in AML, as well as in ALL in two Phase II combination trials, one with the HDAC inhibitor vorinostat (**28**, see also “Histone deacetylases”) together with dexamethasone, and the other with combination chemotherapy. (See Figures 2G and 3B for structures of the compounds discussed in this section.)

#### Anti-apoptotic protein BCL-2

Inhibition of the anti-apoptotic protein BCL-2 sensitizes cancer cells to chemotherapy induced apoptosis (Fulda, 2009). In cell lines and mouse xenografts derived from ALL patients, the BCL-2 antagonist ABT-737 (**21**) demonstrated synergistic cytotoxicity with standard chemotherapy (Kang et al., 2007). Furthermore, in the pediatric pre-clinical testing program the orally bioavailable BCL-2 antagonist navitoclax (**22**) (ABT-263) showed the greatest efficacy against ALL among all the cancer models tested (Lock et al., 2008). A recent study demonstrated cytotoxicity of the BCL-2 antagonist obatoclax (**23**) in a broad range of patient-derived infant ALL cell lines, and synergistic cell killing in combination with chemotherapeutic agents (Urtishak et al., 2013). The clinical effectiveness of obatoclax combined with standard chemotherapy is being tested in a Phase I trial in ALL and AML. (See Figure 2H for structures of the compounds discussed in this section.)

### Transcription

**Figure 5:**
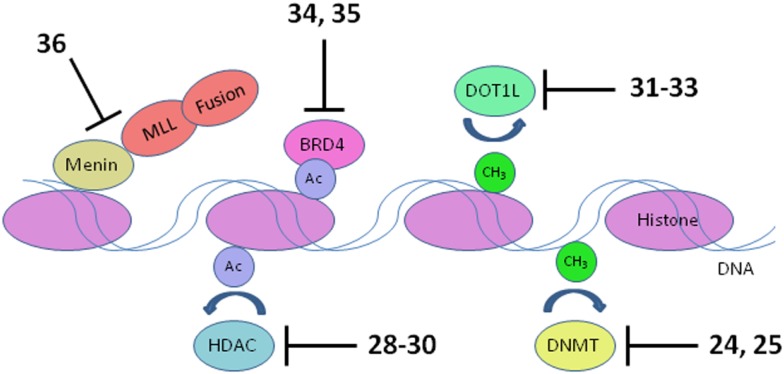
**Transcriptional targets**. Numerous interconnections between targets and other proteins involved in transcriptional activation are omitted for clarity. HDAC, histone deacetylase; DNMT, DNA methyltransferase. Numbers in bold type refer to inhibitors shown inFigure 3.

Inappropriate expression of specific genes caused by dysregulated epigenetic modification appears to be a hallmark of many of the most difficult to treat pediatric leukemias. Early work in this area was focused on inhibition of DNA methyltransferases and histone deacetylases (HDACs), due to the apparent role of these enzymes in preventing expression of key tumor suppressor genes. Much recent activity has been focused on histone methyltransferase, bromodomain, and menin inhibition to suppress transcriptional activation leading to elevated expression of specific oncogenes that block differentiation of hematopoietic progenitors (Figure 5). An epigenetic block of normal differentiation appears to be a key driver of pediatric leukemia harboring chromosomal rearrangements that lead to fusion proteins, notably MLL fusions and NUP98-NSD1 (Deshpande et al., 2012; Hatlen et al., 2012; Popovic and Licht, 2012; Vu et al., 2013).

MLL fusions are the hallmark of an aggressive subtype of pediatric ALL and AML known as mixed lineage leukemia, associated with translocations of the *MLL* gene (Muntean and Hess, 2012). These *MLL* rearrangements are especially prevalent in infant ALL, occurring in 80% of cases, and also in 75% of therapy-related AML. The 5-year survival rate for ALL without *MLL* rearrangement approaches 90%; in stark contrast, infants with ALL harboring *MLL* rearrangements have a very poor 40% survival rate (Robinson et al., 2009; Slany, 2009). The normal role of the MLL protein is to modulate transcriptional elongation through histone methylation. However, rearrangement of the *MLL* gene disrupts the function of MLL by replacement of its C-terminal methyltransferase domain with a fusion protein. Fifty such fusion partners are known, although 5 account for 80% of cases (Huret et al., 2001; Meyer et al., 2006; Krivtsov and Armstrong, 2007). The protein complex recruited by the MLL fusion activates transcription of oncogenes critical for leukemic transformation, notably *HOXA9* and *MEIS1* (Ayton and Cleary, 2003; Faber et al., 2009; Mueller et al., 2009). Following early hematopoietic development, expression of *HOXA9* and *MEIS1* is normally suppressed to allow blood cell differentiation. Activated *HOXA9* blocks differentiation of hematopoietic progenitor cells, which instead acquire stem cell-like character and the capacity for unlimited self renewal (Somervaille and Cleary, 2010). Several components of the transcriptional activation complex in MLL-R leukemia are being targeted: menin, bromodomains, and the methyltransferase DOT1L (Figure 5).

The NUP98-NSD1 fusion protein occurs in a high-risk subtype of AML following rearrangement of the genes *NUP98* (nucleoporin, 98-kd component of nuclear pore complex) and *NSD1* (nuclear receptor-binding SET domain protein 1). Since the first case was identified 12 years ago (Jaju et al., 2001), it has become clear that these genetic lesions are often missed by routine karyotyping. A recent comprehensive study using reverse transcription polymerase chain reaction (RT-PCR) found NUP98-NSD1 in 4–5% of pediatric AML, associated with a grim 4-year event-free survival rate of<10% (Hollink et al., 2011). NSD1 is a histone methyltransferase that regulates gene transcription through methylation of lysine 36 in histone 3 (H3K36) (Morishita and di Luccio, 2011; Wagner and Carpenter, 2012). The methyltransferase activity of NSD1 is retained in the NUP98-NSD1 fusion, and gives rise to abnormally high levels of H3K36 methylation, enforcing activation of transcription of oncogenes such as *HOXA9*. As in MLL-R leukemia, elevated expression of *HOXA9* in AML harboring NUP98-NSD1 blocks differentiation of blood cell progenitors, leading them to acquire the capacity for unlimited self-renewal and malignant transformation (Wang et al., 2007). Abolition of the methyltransferase activity of NUP98-NSD1 by point mutation demonstrated its essential role; the level of H3K36 methylation at the *HoxA9* locus was reduced, and mouse progenitor cells harboring NUP98-NSD1 underwent normal differentiation (Wang et al., 2007). Therefore, inhibition of the methyltransferase activity of NUP98-NSD1 may be a viable therapeutic strategy, although no inhibitors of this fusion have been reported to date.

#### DNA methyltransferases

Elevated levels of DNA methylation lead to impaired expression of tumor suppressor genes and other epigenetic changes that promote precancerous and cancerous conditions (Lyko and Brown, 2005). Two DNA methyltransferase inhibitors have been approved for the treatment of myelodysplastic syndrome: 5-azacytidine (**24**) and decitabine (**25**) (Fandy, 2009). These drugs have been evaluated in clinical trials in AML, and decitabine received European but not US approval in 2012 for treatment of adult AML. Two trials in pediatric AML are pending: a Phase II trial focused on maintenance therapy employing 5-azacytidine in combination with the immunomodulatory biologic sargramostim, and a Phase I trial of decitabine in combination with the HDAC inhibitor AR-42 (**30**, see 2.2.2). (See Figures 3A,B for structures of the compounds discussed in this section.)

#### Histone deacetylases

Acetylation of histones promotes transcription by increasing accessibility of chromatin and exposing acetylated lysine residues as key epigenetic markers that lead to recruitment of RNA polymerase. In the context of cancer, HDACs remove these acetyl modifications, blocking expression of key tumor suppressor genes. Following numerous trials of HDAC inhibitors, two compounds have been approved for the treatment of adult cancers (Giannini et al., 2012). Currently, pediatric leukemia trials are underway to test HDAC inhibitors as single agents and also in combination with DNA methyltransferase inhibitors (Mummery et al., 2011). Vorinostat (**28**) was the first HDAC inhibitor approved for clinical use. It is a relatively simple molecule containing a zinc-binding hydroxamic acid moiety that inhibits a broad spectrum of HDACs. It is in Phase II pediatric testing in ALL and AML. A Phase I trial in ALL and AML is also underway with panobinostat (**29**), another broad-spectrum HDAC inhibitor containing a hydroxamic acid group under development for various adult cancers. A Phase I trial in AML is due to start soon combining the DNA methyltransferase inhibitor decitabine (**25**) with the HDAC inhibitor AR-42 (**30**), which was reported to be superior to other broad-spectrum HDAC inhibitors (Lucas et al., 2010). (See Figures 3A,B for structures of the compounds discussed in this section.)

#### Histone methyltransferase DOT1L

Compelling evidence links recruitment of the histone methyltransferase DOT1L to transcriptional activation of oncogenes such as *HOXA9* in MLL-R leukemia (Bernt and Armstrong, 2011; Zhang et al., 2012). The enzymatic activity of the histone methyltransferase DOT1L has been shown to be essential for MLL-R leukemia in several studies. Conditional knockdown of *Dot1l* in mice harboring the MLL-AF6 fusion inhibited leukemia progression (Deshpande et al., 2013), and the same group demonstrated that *Dot1l* knockout prevented initiation of leukemia harboring MLL-AF10 (Chen et al., 2012). An earlier study showed DOT1L to be essential for *HOXA9* and *MEIS1* expression and the resulting leukemia driven by MLL-AF9 (Nguyen et al., 2011). *DOT1L* knockdown has also been shown to induce apoptosis in cells transformed with a variety of MLL fusions (Chang et al., 2010). Therefore, efforts are underway to develop inhibitors of the enzymatic activity of DOT1L. DOT1L differs structurally from all other histone lysine methyltransferases (HKMTs) in that it does not possess a SET domain comprising the catalytic site. This structural uniqueness in the architecture of the catalytic site facilitates discovery of selective inhibitors. Furthermore, DOT1L is the only enzyme known to methylate lysine 79 on histone 3 (H3K79), reducing the possibility of activation of an alternative cellular pathway to circumvent a block to DOT1L-mediated methylation.

The most significant progress in the development of DOT1L inhibitors has been made using structure-based and mechanistically guided design based on the published X-ray crystallographic structure of the catalytic domain of the enzyme (Min et al., 2003). This approach led to EPZ004777 (**31**), a mimic of the methyl donor substrate *S*-adenosylmethionine (SAM) that inhibits DOT1L with high potency and selectivity. EPZ004777 displayed selective killing of MLL-R leukemia cell lines and suppressed expression of the *HOXA9* and *MEIS1* oncogenes, consistent with the proposed role of the enzyme target. Moreover, despite poor pharmacokinetic properties, 7-day continuous infusion of EPZ004777 resulted in a modest but significant increase in survival in a mouse model of engrafted MLL-R leukemia. The apparent uniqueness of DOT1L’s normal role had earlier raised concerns that inhibitors would prove to be unacceptably toxic, but evidence with EZ004777 suggested otherwise (Daigle et al., 2011). Subsequently, the X-ray crystal structure of EPZ004777 bound to DOT1L was solved, and the structural insights were used to incorporate a bromine substituent in the adenine ring to give SGC0946 (**32**). Compared to EPZ004777, SGC0946 showed an improved *K*_D_ of 60 pM, increased residence time on the enzyme due to a slower dissociation rate, and improved cell permeability, leading to greater efficacy in reducing intracellular H3K79 methylation (Yu et al., 2012).

Identification of a hydrophobic pocket present in the SAM binding site of DOT1L but absent in all other histone methyltransferases led to the design of a SAM mimic with two key features: a benzyl moiety appended to the adenine ring to impart selective binding, and replacement of the *S*-methyl group with an *N*-iodoethyl group for irreversible covalent attachment to the ε-amino group in lysine 79 of histone 3 (**33**). This compound gave an IC_50_ of 110 nM and displayed>1000-fold selectivity for DOT1L over other histone methyltransferases. However, the mechanism of action of the compound was not studied to determine whether it reacted with the lysine-containing substrate as predicted (Yao et al., 2011). A follow-up publication reported that 33 and other compounds containing a reactive *N*-iodoethyl group did not show the desired selective killing of MLL-R leukemia cells, so SAM analogs containing other *S*-methyl group replacements were synthesized. The most potent of these were analogs of EPZ004777 comprising the native adenine ring appended with N^6^ substituents. Although some cellular activity was observed in MLL-R leukemia cell lines, none of the compounds appeared to offer advantages over EPZ00477 itself (Anglin et al., 2012).

Recently the possibility has arisen that MLL-R leukemia may be targeted by blocking ubiquitinylation of histone 2B, preventing activation of DOT1L. Following a demonstration that the activity of DOT1L is markedly enhanced by interaction of the enzyme with ubiquitinylated histone 2B (McGinty et al., 2008, 2009), it has now been reported that proliferation of MLL-R leukemia cells was suppressed by knockdown of the ubiquitin ligase RNF20 that ubiquitinylates histone 2B (Wang et al., 2013). (See Figure 3C for structures of the compounds discussed in this section.)

#### Bromodomains

BRD4 is a member of the BET (bromodomain and extra-terminal domain) family of proteins that binds to acetylated lysine in histones via a structural motif known as a bromodomain. In several cancers including acute leukemia, the resulting activation of transcriptional elongation promotes the expression of growth-promoting oncogenes (Deshpande et al., 2012; Popovic and Licht, 2012). An siRNA screen in AML cells revealed that inhibition of BRD4 led to cell cycle arrest and apoptosis. Structure-based design based on the X-ray crystal structure of the bromodomain in BRD4 led to (+)-JQ1 (**34**), a potent and selective inhibitor of binding of acetylated lysine (Filippakopoulos et al., 2010). (+)-JQ1 suppressed proliferation of AML cells in culture and also extended survival in a mouse model of AML (Zuber et al., 2011). Subsequently (+)-JQ1 was shown to be cytotoxic to B-cell ALL cell lines, especially those harboring rearrangements of CRLF2. CRLF2 heterodimerizes with the IL7 receptor (IL7R) and drives proliferation and resistance to apoptosis. Strikingly, the genes most strongly downregulated by (+)-JQ1 were *MYC* and *IL7R* (Ott et al., 2012). (+)-JQ1 also suppressed growth and induced apoptosis in primary cells from patients with freshly diagnosed and relapsed AML. Based on these promising results (+)-JQ1 will be advanced to clinical trials (Herrmann et al., 2012). Another bromodomain inhibitor, I-BET151 (**35**), optimized for selective binding to BRD3/4 and good *in vivo* pharmacokinetics, gave a promising increase in survival in two mouse models of MLL-R leukemia (Dawson et al., 2011). The role of BRD4 in activation of transcription became clearer and potentially more significant with the discovery that it possesses protein kinase activity. BRD4 phosphorylates serine 2 on RNA polymerase II and thereby promotes transcriptional initiation and elongation (Devaiah et al., 2012). (See Figure 3D for structures of the compounds discussed in this section.)

#### Menin and other proteins interacting with MLL fusions

Another approach to disruption of transcriptional activation in MLL-R leukemia is to inhibit the interaction of MLL and menin. Menin appears to be a central hub that interacts with MLL fusions as well as wild type MLL and various other proteins to promote leukemia (Thiel et al., 2012). High-throughput screening for inhibitors of binding of an MLL-derived peptide to menin identified hits that were optimized for potency, leading to MI-2 (**36**). MI-2 was shown to disrupt the intracellular interaction of menin and MLL-AF9 and arrest cell growth in MLL-R leukemia cells, albeit with rather modest potency in the low micromolar range. Moreover, MI-2 blocked the transforming ability of MLL-AF9, suppressed the expression of the *HOXA9* and *MEIS1* oncogenes, and induced hematopoietic differentiation. No animal data has been reported so far; the intention is to optimize the inhibitors further to enable assessment of their therapeutic potential (Grembecka et al., 2012).

In addition to menin, various transcription factors and other proteins interact with MLL fusions to form an activation complex that promotes transcriptional elongation (Slany, 2009). In principal, several protein–protein interactions in this complex could be viable targets for disruption. One interaction that has been validated as a target with a peptide, and is being screened for small-molecule inhibitors, is the binding of MLL-AF4 to the transcription factor AF9 (Watson et al., 2013). A direct AF4–AF9 interaction has been demonstrated (Erfurth et al., 2004), and shown to be essential for the survival of leukemia cell lines containing MLL-AF4 and MLL-AF9 fusions (Srinivasan et al., 2004). Yeast two-hybrid assays identified the minimal amino acid sequence in AF4 required for binding to AF9, and a cell-permeable peptide derived from this sequence was shown to disrupt intracellular AF4–AF9 binding. Moreover, this AF4-derived peptide killed cells harboring MLL-AF4 and MLL-AF9 fusions but had no effect on normal hematopoietic progenitor cells (Srinivasan et al., 2004). (See Figure 3E for structure of the compound discussed in this section.)

### Chemokine receptors and stem cell homing

Antagonists of the chemokine receptor CXCR4 could be effective as chemosensitizing agents by preventing homing of LSCs to the bone marrow. LSCs residing in the bone marrow evade treatment by chemotherapeutic agents, resulting in resistance and relapse (Attar, 2012; Peled and Tavor, 2013). Plerixafor (**37**) is an antagonist of the CXCR4 chemokine receptor that has been FDA approved since 2008 for hematopoietic stem cell mobilization prior to transplantation in non-Hodgkin’s lymphoma and multiple myeloma. Encouraging results were reported recently from a Phase I trial of plerixafor in combination with cytarabine and etoposide in adult AML (Uy et al., 2012) and a pediatric ALL and AML trial is now underway. A recent report (Sison et al., 2013) shows that CXCR4 antagonists may also synergize with other targeted therapies; in a mouse model of infant MLL-R ALL, plerixafor markedly enhanced the efficacy of the FLT3 inhibitor lestaurtinib (**4**). (See Figures 2B and 3F for structures of the compounds discussed in this section.)

## Concluding Remarks

Based on a steady increase in understanding of the cellular mechanisms operating in high-risk pediatric leukemias, there is an encouraging level of activity aimed at the discovery of targeted therapies. Some of the molecular targets, such as protein kinases, are already well established in adult cancers. In other cases, pediatric leukemia research has led to novel targets that had not previously attracted attention; a notable example is the histone methyltransferase DOT1L. Given the number and diversity of new chemical entities currently in pre-clinical and clinical development for high-risk pediatric ALL and AML, the prospects are good for significantly improved outcomes for children afflicted with these cancers.

## Conflict of Interest Statement

The authors declare that the research was conducted in the absence of any commercial or financial relationships that could be construed as a potential conflict of interest.
